# Treatment of Stricture of the Urethra by Rapid and Free Dilatation

**Published:** 1853-08

**Authors:** 


ART. III.— *Treatment of Stricture of the Urethra by Rapid and Free Dilatation. Illustrated with cases.* By PAUL F. EVE, M. D., Prof. of Surgery in the University of Nashville.

Prof. Eve is not a prolific, certainly not a prolix writer. When he does write he tells in a plain, condensed, and scientific manner, whatever he has to say. And this little *brochure,* like everything from his pen, has a merit which may not be estimated by its brevity, or the modest style in which it is put forth. In a few effective sentences he proves that our previous treatment of stricture was not radical, but only palliative—that the liability to a renewed coarctation amounts almost to certainty. He then proposes the treatment of M. Béniqué, by very rapid dilatation. But we cannot do better justice to Dr. Eve, than by segregating some extracts from his treatise. On page 8 he says:

"I have not seen M. Béniqué's 'Reflections and Observations on Strictures of the Urethra,' but my own have brought me to the conclusion, that the universal practice of regulating or measuring the *size* of the dilating instrument, by the *orifice* of the urethra, has been unfortunate, and lead to the general result of this plan, failing to remove the complaint. The diameter of this excretory canal, it is well known, is far from being uniform, neither is it cylindrical. All agree that the orifice is the most restricted. This may be assumed at 2 or 2½ lines in diameter, while that of the urethra itself measures 3½ to 4 lines. It is a closed tube, but if the external meatus be incised

---

or gradually dilated, it will readily admit an instrument of the caliber last mentioned. Like all openings of mucous surfaces, this one is naturally contracted, and it requires but an experiment, simply to enlarge it, to convince any one who may doubt that its diameter is not considerably increased beyond it. The well known practice of lithotritists to introduce their large instruments by incising the meatus, is proof positive of this fact, which should be more generally known, viz., that this passage is much more capacious than its mouth. How then can a bougie which fills only the orifice of the urethra dilate a portion of the canal that is primitively much wider; or still more difficult, restore a contracted part to its original size? To this there is a physical impossibility. Now by a large sized bougie, or catheter, employed against Stricture is meant one that fits the external opening and not the passage beyond; therefore, must it necessarily follow that dilatation as commonly applied, while it may relieve for a time, cannot cure the affection. But let an instrument measuring 4, 4½, or 5 lines in diameter as recommended by Béniqué, be cautiously, gradually insinuated into a restricted portion of the urethra, by incising or dilating its orifice; the impracticability of the operation is removed, the disease can be reached and may then be cured.

"Since writing the above, I find from the last number of the Philadelphia Medical and Surgical Journal, that the late celebrated Dr. Physick, entertained similar views to those here expressed.

"In recommending full and rapid dilatation in the treatment of Strictures, and contending that it is almost the only certain way of effectually curing them, I am at the same time decidedly opposed to the forced or sudden introduction of instruments into the bladder through the urethra. This practice attempted to be established by M. Mayor, of Lausanne, Switzerland, a few years ago, is utterly opposed. It has only been after other means have failed that forcible pressure against a stricture was resorted to, in order to relieve a distended bladder: but in every instance violence was avoided and great prudence exercised. In catheterism every movement should be made with caution and skill, perseveringly yet patiently, that the instrument may insinuate itself, be drawn as it were into the bladder by an invisible yet tangible suction power, and not suddenly, violently or forcibly thrust into it. And while I agree that in every case when the instrument excites irritation it should be removed, still it must be manifest that the more rapidly a restricted portion of the urethra is restored to its original size without causing pain or inflammation, the more economically, in every respect, will be the cure. But as this disease lies concealed in a passage wider than the orifice leading to it, free access to its location can only be had by enlarging this opening. In cases of rupture of the urethra or of its division by an operation, dilatation is still considered essential to overcome the tendence to contraction and coarctation of cicatrices. The lymph effused under these circumstances soon becomes converted to mucous membrane, and to expand it occasionally is certainly the proper way to adapt and mould it to the healthy functions of this canal; and experience has proven, that the gentle passing of a smooth instrument does not materially interfere with the extension of the cylindrical epithelium over a wounded surface. In advocating a new method of dilatation to cure stricture, let it then be understood that its cautious and gradual application, though it be rapid and ample, is none the less insisted upon; and sudden or forcible measures honestly condemned.

"It is admitted that these cases are defective, especially in regard to time, as confirmatory of the treatment of urethral stricture now proposed and herein recommended; but they are the most favorable which have occurred in the brief space since my return from Europe, and are presented to sustain the following new positions:

"1st. That while dilatation is the proper treatment for Stricture of the Urethra, *this* has hitherto failed to effect a cure, because in the ordinary mode of applying it, *the seat of the disease has not specially been acted upon by the dilating instruments.*

"2d. To cure Stricture, the *orifice* of the urethra must be so enlarged that the *canal beyond it may be dilated to its original size,* which we ought to recollect is about *twice* that of the opening leading to it. Instead, therefore, of being satisfied with introducing bougies of *two lines in diameter* through a restricted portion, they should measure *four to five lines* in thickness.

"3d. *There is no necessity to confine a patient to bed* in treating Stricture; *once an instrument has been introduced, it has done all it can to expand the passage and should be withdrawn, that others larger in size may be immediately substituted.* While this process ought to be cautiously and very gradually conducted, still the more rapidly and freely it can be applied, provided no pain is excited, the sooner the disease will be removed.

4th. By this method Strictures may be *permanently cured in a few days, without suffering, inconvenience, or exposure to serious consequences.*"

